# The X-Rays

**Published:** 1912-05-25

**Authors:** Alfred C. Norman

**Affiliations:** House Surgeon at Sunderland and Durham County Eye Infirmary, Sunderland.


### ELECTRICITY IN MODERN MEDICINE.*

### XII.—The X-Rays.

By ALFRED C. NORMAN, M.D. Edin., House Surgeon at Sunderland and Durham County Eye Infirmary, Sunderland.

### Efficiency of the Interrupter.

Since the current which we send through the $x$-ray tube is a series of unidirectional discharges induced at "break" of the primary circuit (the "make" discharges being suppressed), and since each discharge produces a certain quantity of $x$-rays, it would seem that the oftener we break the primary circuit per second the more $x$-rays shall we obtain from the tube in a given time. And this is found to be the case, *provided that the intensity of the discharges are the same in each instance.* But, if we run our interrupter so fast that the primary circuit is broken before it has had time to reach its full strength, and before the magnetic field has arrived at its full degree of saturation, we shall be producing discharges of weaker intensity from the secondary than we can obtain by running the interrupter more slowly. The total amount of $x$-rays produced may be the same (the loss in intensity being balanced by the increased number of discharges per second), but there is a greater tendency to produce reverse current with all its attendant disadvantages.

For these reasons the writer is inclined to doubt the wisdom of makers who supply a coil with a small iron core so that it shall lose and regain its magnetism very quickly and thus be available for use with a very rapid interrupter. The individual discharges must necessarily be smaller, so that there is not much to gain in that way, and there is a good deal to lose in extra wear and tear on the tubes.

To produce a steady discharge from the tube (and a steady fluorescence on the screen) it is necessary to have at least thirty to forty interruptions per second, and, generally speaking, there is no advantage to be gained in going beyond about eighty interruptions per second.

*Duration of contact* is another important factor in the working of an interrupter. In a complete cycle there is a certain fractional period of time during which the primary current is flowing; this corresponds to the period during which the metallic segment is dipping into the mercury and is known as the *duration of contact*; the rest of the cycle, in which the current is not flowing, we may term the *period of recovery.* Now, although each cycle occupies less than one-fortieth of a second, there is a certain ratio which varies with every coil and with every rate of interruption, between the duration of contact and the resting period which gives the most satisfactory output from the $x$-ray tube.

All the best interrupters have a handle by means of which the duration of contact can be adjusted while the apparatus is working, and there should also be a rheostat on the switchboard for controlling the speed of the motor. With the aid of these adjuncts it becomes a simple matter to vary the rate of interruption and the duration of contact so as to obtain the best results from the $x$-ray tube. The most satisfactory plan is to use a milliampere meter in the secondary circuit and, with a given current in the primary, to vary the rate of interruption and the duration of contact until the milliampere meter registers the highest amount of current through the tube. If this is too much for the tube the primary current must then be reduced and the rate and duration readjusted. The golden rule is—*work with the minimum rate of interruption that will give the maximum discharge through the tube.* (We shall see later that the milliampere meter reading is really a resultant of milliamperage and frequency.)

### Care of the Interrupter.

All types of mercury interrupter have to be cleaned from time to time, because some of the mercury is oxidised as a result of the sparking, and some of it is churned into a finely divided state, forming a sort of mud, and this fouls the contact and spoils the sharpness of the break. If the $x$-ray outfit begins to give poor results after some weeks or months of use, one should suspect that the mercury break is at fault and should have it cleaned out at once. This is a messy business at best, though it is not so bad with a coal-gas as with a liquid di-electric. The mercury should be taken out and washed under a stream of hot water until it is free from oxide; it must then be thoroughly dried (by pieces of blotting paper) and returned to its place in the interrupter, a little more being added to bring it up to its original amount. The di-electric should of course be renewed.

The centrifugal interrupter requires cleaning less often than many others, because the centrifugal action carries the dross away from the contacts. This type costs about £10; a Mackenzie Davidson interrupter about £6.

### THE X-RAY TUBE.

Before considering a modern $x$-ray focus tube it will be necessary to review briefly some of the physical phenomena set up by the discharge of high voltage interrupted currents through a tube that has been exhausted of air to form a partial vacuum.

Let us take a glass bulb or tube about 6 inches in diameter into opposite walls of which two platinum wire electrodes have been fused so that a

* Previous articles appeared on Nov. 11, 25, Dec. 9, 30, Jan. 13, 27, Feb. 17, March 9, 30, April 20, May 4.

free end of each wire projects into the tube and is separated from the opposite wire by a space of about 3 inches. At one end there is a nozzle, which can be connected with a pump for exhausting the tube of air. If we connect the two wires of such a tube with the discharging pillars of an induction coil the nature of the electrical discharge through the tube will be greatly modified as we gradually withdraw the air by means of an exhaust-pump. Let us take three stages:

(It should here be noted that the electrode of the tube connected with the positive pillar of the coil is always called the anode, and that connected with the negative pillar the cathode.)

1. If we turn on the current before any air has been removed we find that the discharge jumps across the air-gap between the anode and cathode inside the tube just as it would in the open air, making a crackling noise and giving rise to a series of sparks. The resistance of the tube to the passage of electricity is, of course, exactly equivalent to the resistance of the air-gap between its electrodes.

2. If we now exhaust the tube, by means of the air pump, to about the $\frac{1}{1000}$ part of an atmosphere it is known as a Geissler tube, and the character of the discharge which an induction coil passes through it is entirely changed. Its resistance to the passage of electricity is considerably less, so that the current passes through the 3-inch space between the electrodes much more easily than it did when they were surrounded by air at atmospheric pressure. The passage of the current is no longer accompanied by sparks or noise, but by a beautiful stream of blue light which extends across the tube from the cathode to the anode. This stream is easily deflected from its path (by holding one's finger to the tube for instance), and where it touches the glass wall of the tube a yellowish fluorescence is excited in the glass. The stream of blue light is termed the "cathode beam"; it is really a fluorescence excited in the rarefied gas by the passage of an invisible stream of particles from the cathode known as the "cathode rays." The yellow fluorescence of the glass wall of the tube is also caused by the cathode rays. These rays are the progenitors of the *x*-rays, and will have to be considered at greater length.

3. If we exhaust the tube to less than the $\frac{1}{1000000}$ part of an atmosphere it is called a Crookes tube (in honour of Sir William Crookes, who did so much pioneer work with high-vacuum tubes). The resistance of such a tube to the passage of electricity is very much greater than was the case with the Geissler tube; in fact it may even be considerably greater than was the original resistance of the tube when filled with air at atmospheric pressure.

Crookes tubes and *x*-ray tubes are always classified according to their electrical resistance rather than to their degree of exhaustion, because the former is so much more easily measured from day to day. It is an easy matter to connect an *x*-ray tube, in parallel with a variable spark-gap, to the induction coil and to notice the length of air-gap the current will spark across rather than pass through the *x*-ray tube. This is termed the *equivalent spark length* of the tube, and is really a measure of its resistance to the passage of a current. It could be expressed in ohms, remembering that about 15,000 volts are required to force a current across an air-gap of 1 inch, but this would be of little value because electrical discharges through vacua do not conform to Ohm's law.

Crookes tubes of from 2 to 8 inches equivalent spark are capable of generating *x*-rays which pass out of the tube in useful quantities. A tube exhausted to about the $\frac{1}{1000000}$ part of an atmosphere has a resistance equivalent to about a 2-inch air-gap. A tube very considerably more exhausted would have a resistance equivalent to perhaps 8 inches; while a tube which is as nearly as possible a complete vacuum would not transmit any current at all.

The following is a possible explanation of the variations in resistance which a tube undergoes as its vacuum increases. We might suppose that electricity is carried across the space in the tube between cathode and anode by material particles (ions) of the gases which remain in the tube. Before the tube has been exhausted at all the particles are so crowded together that their excursions of movement are comparatively limited, they convey their burden with some difficulty, and so the tube has a certain resistance to the passage of electricity. When we exhaust the tube to the Geissler degree (about 1/1,000 of an atmosphere), there are still plenty of particles to carry the current across the tube, but they now have plenty of room, so to speak, in which to move rapidly; consequently they transmit the current with ease and the tube is found to be of low resistance. Next, when the tube is exhausted to the Crookes degree of vacuum (1/1,000,000 of an atmosphere and upwards), there are not enough particles left to carry the current, and so we say the resistance of the tube has increased. Finally, when the tube is exhausted practically to a complete vacuum, there are no particles of air left to carry the current, and so it becomes a non-conductor. This was the end of many good tubes before they were fitted with regulating devices.

The above explanation should not be taken too literally, but it will serve to keep one in mind of the sequence of events which follow as the vacuum of a tube increases. We shall see later that the greater the exhaustion of a tube the more penetrating are the *x*-rays produced.

But to return to the Crookes tube and the cathode rays. When current is passed through a Crookes tube with simple wire electrodes the phenomena are not very striking to the eye, though they are of extreme scientific interest and their investigation led to the discovery of the *x*-rays. A stream of cathode rays is travelling with lightning-like rapidity from every part of the surface of the cathode, but we cannot see them; *x*-rays are being discharged wherever the cathode stream strikes the glass, and these are also invisible; all that we do see is a little greenish fluorescence where the cathode stream strikes the glass, for there is not enough air in the tube to produce by fluorescence a visible "cathode beam." To gain a proper understanding of the working of an *x*-ray tube it will next be necessary to consider some of the properties of the cathode rays.

(*To be continued.*)

